# Severn School of Psychiatry education fellowships: a new way to promote educational practice and research

**DOI:** 10.1192/pb.bp.113.046052

**Published:** 2015-04

**Authors:** Rob Macpherson, Sherlie Arulanandam, Guy Undrill, Simon Atkinson, Steve Arnott, Sian Hughes, Hannah Toogood, Karl Scheeres, Luciana Matone

**Affiliations:** 1 Health Education South West, Bristol; 2 Bristol Royal Infirmary; 3 The Crisis Resolution and Home Treatment Team, Cheltenham; 4 Teaching Learning for Health Professionals, Bristol University; 5 AWP NHS Trust, Bristol; 6 South Glos Intensive Support Team, Bristol; 7 New Friends Hall, Bristol; 8 Melksham Community Hospital

## Abstract

This paper describes a model of training in leadership and project management skills for advanced trainees, using educational projects within the Severn School of Psychiatry. Fellowships lasting 1 year have been developed to enable trainees, working with a senior consultant trainer associated with the School of Psychiatry, to support important new educational initiatives. Linkage with the local university training and learning for health professionals research module has provided academic support for the trainees and the projects. Four examples for the first year of the programme are described and feedback from structured interviews with participants is presented. The development of the fellowships appears to have had wider benefits, in developing educational faculty in the School of Psychiatry and the trainees involved have had opportunities to extend their project management and leadership skills. The fellowship programme is continuing to develop, based on feedback from its first successful year.

## Background

To increase the range of opportunities and quality of education within the Severn School of Psychiatry, we have in recent years developed a range of projects across the School. Although there is a strong group of trainers, training programme directors, tutors and directors of medical education (DME) who provide leadership, there is limited time within job planned allowances across the senior trainer group to permit extensive work within projects. Advanced trainees need to develop leadership skills and we felt an obvious vehicle to promote this was through co-leadership of educational projects. There are opportunities across the School for projects in areas of educational theory, quality assurance, review of educational processes, innovation and new methods of training/assessment. The Royal College of Psychiatrists’ curricula for advanced training in each specialty contain specific leadership domains and recommendations for training in this area, but a survey of advanced trainees^1^ showed that many questioned how prepared they felt for the role of consultant. The survey indicated they felt well prepared for the clinical role, but less prepared for leading, designing and changing services. There was felt to be a need for higher training to engage with the entirety of the new consultant role.

A business internship model, which now operates across all areas of the Severn School of Psychiatry, has provided opportunities for project-based management training. Similar to the paired learning initiative described elsewhere,^2^ this has had wider benefits for the trainee population and also to the organisations where trainees have had opportunities to work on important trust management projects. We noted that as well as preparing trainees for the management aspects of their new consultant roles, it is important to prepare them fully for the educational role as the majority will become trainers. A smaller group will go on to develop higher-level training responsibilities, such as tutor, training programme directors, DME or head of school role. Project management has the potential to facilitate development across a wide range of areas because of its multifaceted nature and the requirement to work across a variety of interfaces.

## Programme description

The project aimed to offer four educational fellowships over 1 year, allocated through competition. A number of senior faculty members took a lead role in overseeing the programme: the head of the School of Psychiatry (R.M.); the lead for the local MRCPsych core course (S.H.); the regional advisor (G.U.); the lead from the local university training and learning for healthcare professionals department (S.Atk.); and a local director of medical education (S.Arn.). The fellowships were made available by email invitation to all advanced trainees (approximately 50). Trainees were invited to submit an application to undertake a fellowship, through writing a 500-word statement, expressing their interest in this and their key relevant background skills. An assessment framework was developed to rank submissions to decide on appointment and fellows were appointed in the spring 2012, to start their fellowship from August of that year.

Fellowships lasted a minimum of 1 year, with the fellows meeting monthly for peer support, together with project leads and with educational supervision from the teaching and learning for healthcare professionals (TLHP) team. It was expected that trainees use non-clinical special interest time for the project. Each project was overseen and co-led by a consultant with a specific, related educational role. We suggested that the educational fellow worked with a core trainee in his or her trust, aiming to involve a wider number of trainees, to provide an interesting and attractive opportunity for the core trainee and also to give opportunities for the fellows to give academic/project supervision, viewing the core trainee as a research assistant in this context. Depending on commitments, core trainees were welcome to attend the fellowship supervision sessions.

The work required was expected to vary across the different projects, but some common requirements were set out: literature review and summary of relevant evidence; liaison with key stakeholders including service user and carer groups; liaison with the trainee group; development of option appraisals; preparation of reports; presentation of material to the wider educational community; and dissemination through deanery and regional/national training forums.

The fellows were automatically enrolled on the TLHP research methods module, with an expectation that each fellow attend two study days and complete assignments, based on their fellowship project. On successful completion of the course they would be awarded 20 credits at Masters level (approximately one-third of the Masters course) and were able to take this credit into any further work in this area. The costs of the module (approximately £1200) were supported by the Deanery, other than for one fellow who was funded directly by the DME. Meetings of the project group were held in the Deanery and any costs for specific activities related to projects were considered by the head of School, in collaboration with the school of psychiatry manager in the Deanery. The programme was managed through the School of Psychiatry and issues arising reported to the board and wider deanery colleagues as necessary. Further governance support was obtained as needed through the Severn Deanery Executive team, who supported the project.

## Projects overview

### Training across boundaries (TAB)

This project, led by R.M. and S.Aru., explored the development of training links between core psychiatry and medical/general practice posts. It aimed to provide opportunities for exposure to different medical specialties. Although this has been recommended in the psychiatry training literature, there were no examples in the literature that we could find to describe this. The project focused on core training years two and three across two mental health trusts. The project was carried out in a single 6-month period from February to July 2013. Trainees were encouraged to maintain a reflective log and ideally complete a workplace-based assessment in their project. Up to six TAB sessions were encouraged during the 6-month placement. The project was relatively successful, 12 trainees undertaking a TAB placement in the time specified. Placements included general practice, neurology, paediatrics, endocrinology, emergency medicine and neuroimaging. The programme was more trainee-led than expected. Feedback suggested trainees had a range of valued, positive training experiences and this project will be written up elsewhere.

### Supervision project

This project, led by S.Aru. and L.M., focused on exploration of educational supervision. It was noted that despite supervision being a central aspect of psychiatric training, the literature was limited regarding the activity occurring within supervision sessions. Nine structured interviews of advanced trainees were carried out by L.M. These focused on experiences of supervision and were transcribed, then subjected to thematic analysis, with the expectation of disseminating the results.

### Communication skills training project

This project was led by G.U. and H.T. The project noted a paucity of literature relating to the evidence around communication skills appropriate to basic psychiatric training. A programme of development of the communication skills training within the core psychiatric trainees’ course was undertaken. This included use of videos and more systematic, structured feedback. This was developed into more refined skills assessment using simulation and is now being further developed with the use of a software package to formally rate aspects of communication skills and provide structured feedback to the interviewer.

### Developing knowledge and skills among core trainees

This project was led by S.H. and K.S. The project started from an awareness of local and national difficulties in knowledge, skills and competence within the core psychiatric trainee group. It was noted that many trainees had problems passing the College clinical exam, despite successful completion of clinical competence requirements in placements. The project identified a range of different interventions potentially of use to help core trainees. These included a mock clinical assessment of skills and competencies (CASC) exam, additional communication skills training, cultural awareness training, cognitive assessment and targeted skills development. The project is being further developed in a second round of educational fellowships.

## Results

In the first year of the fellowship there were 12 supervision sessions. Trainee attendance ranged from 5 to 11, in total 34 attendances across the year. Consultant attendance ranged from 9 to 11, total 40 attendances across the year.

### Feedback from educational fellows

The educational fellows reported that the administration of the programme was effective and they felt well engaged in their projects and the supervision programme. There was some concern about the way trainees were linked to individual projects and the potential for trainee choice about projects undertaken. However, it was noted that within the projects there was substantial choice and multiple opportunities for individual development. Supervision was found to be helpful, particularly with regards to the attendance by the TLHP team. The network of supervision provided a useful prompt to ensure that projects did not stagnate and was found to be interesting, although in some cases the individual’s supervision with project supervisors was felt to be more helpful in terms of project design and problem-solving.

Communication outside supervision ranged from two to four times per month, with trainees feeling they had appropriate levels of support. In terms of challenges encountered during the project, the fellows reported some difficulties in managing the project within time restraints and in two cases a risk of the project escalating into an unmanageable, larger scale research project. The trainees felt that more specific input from an educational specialist could have been valuable across the programme and that greater attention could have been paid to educational and management theory alongside the practical experiences gained. The two trainees who completed the TLHP research module found it helpful and highly relevant to their involvement in the fellowship. One trainee planned to attend the course at a later stage.

Trainees reported a range of different opportunities arising from their involvement. These included the development of teaching, mentoring and education research skills, as well as softer benefits such as increased confidence and networking with research experts. Trainees also gained understanding of the challenges of project management and dealing with resistance. Issues around data protection and consent were sometimes difficult and required negotiation with senior colleagues in the Deanery. Liaison across different disciplines was valued and one trainee reported that using formal project management systems such as a Gantt chart had been valuable. Two of the trainees reported that the fellowship had a significant impact in the development of leadership skills and enabling reflection on leadership style. A range of different skills including transactional, participative approaches were required and it was noted that developing a non-hierarchical, inclusive style of working with trainees and others had been important. It was felt that attention to the development of leadership skills could be helpful in future fellowship programmes. All four trainees recommended the fellowship programme to other trainees and there were a range of other comments including the suggestion that this was the most helpful leadership/management training experience obtained so far.

### Feedback from the consultant leads

This was obtained through a similar format. Advice from other consultant colleagues and the TLHP team within supervision sessions was found to be helpful, as was group support and input from trainees. It was noted that working with bright trainees who were highly motivated and interested in education enabled the development of a wider educational faculty. The consultant group noted that there had been some specific problems around transcription and information technology that had hindered the completion of some projects. It was noted that in some cases there was a process of continuing improvement/development, meaning that the completion of one aspect of a project would open up further developments. The consultants particularly valued the opportunity to work closely with senior trainees and to develop a major project. It was noted that issues around leadership and project management were centrally involved throughout the project and this provided many opportunities for training and learning. The fellowships also provided opportunities for creative development that was not constrained by bureaucracy and seemed to develop the skills of trainees who were likely to become the senior trainers of the future. There was significant learning around the area of leadership and project management in the consultant group also, particularly around the balance between guidance and instruction/direct facilitation. It was felt that ideally trainees should be allowed to develop their skills by testing their own ideas out. It was noted that coaching skills, rather than supervision or line management was most helpful in this context. The consultants all recommended the fellowship programme to other trainees and to other consultant colleagues, particularly those involved in a senior educational role.

## Discussion

This paper presents a model for training advanced trainees in leadership, education and management through co-development of educational projects with senior educationalists in a school of psychiatry. It was apparent that, as reported in other educational literature,^3^ effective leadership of the projects was associated with being proactive and fully engaged with the group affected by the project. The educational fellow and paired consultant needed to integrate a clear project focus and vision, with effective implementation, for projects to progress effectively. Five factors have been associated with effective leadership^4^ (Box 1) and recognition of these featured frequently in feedback from consultants and educational fellows.

**Box 1** Factors associated with effective leadership^4^

- Modelling the way – leading by example in a manner that is consistent with leader’s stated values.
- Inspiring a shared vision – developing a compelling vision of the future and enlisting the commitment of others.
- Challenging the process – being on the look-out for opportunities to improve the organisation and being prepared to experiment.
- Enabling others to act – promoting collaborative working; empowering others; building trust.
- Encouraging the heart – recognising individuals’ contributions; celebrating accomplishments.

Other feedback noted that a key process in this project was the development of senior educational faculty (including the advanced trainees) and the development of a collegiate culture based on the principles of coaching and mentoring, which can have benefits for educational satisfaction, academic and personal development.^5^ There were obvious benefits for senior faculty in participation in this work and an aim of this project was to facilitate project management across the School, giving opportunities for the consultants to complete work that otherwise may not have been feasible. For the trainees, the project enabled holistic development, encompassing academic learning and the development of skills such as problem-solving and analysis. There is a need for training organisations to provide a portfolio of learning opportunities and resources, to facilitate the development of management and leadership skills among senior trainees.

The joint supervision sessions were valued by all participants. Others have noted^6^ that it is the process of bringing many constituencies into debates that facilitates transformation, rather than restricting the process to a small number of consensual voices. Such ideas fit naturally with theory around ‘sense making’, which emphasises the role, after the initial impetus has been set by leaders, of inclusive and widespread conversations and reflection, to explore new possibilities and the emerging pattern of changes.

### Future plans

It is hoped that a number of this first phase of projects will produce publications and be presented at regional and College forums. The fellowship programme is now in its second year and has recruited four new trainees into new projects, with two new consultant leaders. The fellows from year one are invited to continue to attend supervision sessions as are the two consultants no longer actively working on projects. Feedback from year one was used to plan for greater choice of projects for trainees, increased academic and theory input to supervision sessions and the involvement in the TLHP module has been timed to fit better with the project timeline.
